# Presynaptic Self-Depression at Developing Neocortical Synapses

**DOI:** 10.1016/j.neuron.2012.10.035

**Published:** 2013-01-09

**Authors:** Antonio Rodríguez-Moreno, Ana González-Rueda, Abhishek Banerjee, A. Louise Upton, Michael T. Craig, Ole Paulsen

**Affiliations:** 1 Department of Physiology, Development and Neuroscience, University of Cambridge, Cambridge CB2 3EG, UK; 2 Department of Physiology, Anatomy and Genetics, University of Oxford, Oxford OX1 3PT, UK; 3 Department of Physiology, Anatomy and Cellular Biology, University Pablo de Olavide, 41013 Seville, Spain

## Abstract

A central tenet of most theories of synaptic modification during cortical development is that correlated activity drives plasticity in synaptically connected neurons. Unexpectedly, however, using sensory-evoked activity patterns recorded from the developing mouse cortex in vivo, the synaptic learning rule that we uncover here relies solely on the presynaptic neuron. A burst of three presynaptic spikes followed, within a restricted time window, by a single presynaptic spike induces robust long-term depression (LTD) at developing layer 4 to layer 2/3 synapses. This presynaptic spike pattern-dependent LTD (p-LTD) can be induced by individual presynaptic layer 4 cells, requires presynaptic NMDA receptors and calcineurin, and is expressed presynaptically. However, in contrast to spike timing-dependent LTD, p-LTD is independent of postsynaptic and astroglial signaling. This spike pattern-dependent learning rule complements timing-based rules and is likely to play a role in the pruning of synaptic input during cortical development.

## Highlights

► Natural spike patterns in layer 4 neurons induce LTD at downstream synapses ► Spike pattern-dependent LTD can be induced in individual presynaptic neurons ► Spike pattern-dependent LTD requires presynaptic NMDA receptors and calcineurin ► Spike pattern-dependent LTD is independent of postsynaptic and astroglial signaling

## Teaser

Using natural spike patterns recorded from cortical layer 4 neurons in vivo, Rodríguez-Moreno et al. uncover a new spike pattern-dependent synaptic learning rule. They find that individual presynaptic neurons can drive NMDA receptor-dependent synaptic depression without a requirement for postsynaptic activity.

## Introduction

Activity-dependent synaptic plasticity plays a central role in the refinement of synaptic connections in the cerebral cortex (Feldman and Brecht, 2005; Caporale and Dan, 2008). Correlated activity between pre- and postsynaptic neurons is believed to be important in driving such synaptic modifications, as first famously captured by Donald Hebb’s neurophysiological postulate, “When an axon of cell A is near enough to excite a cell B and repeatedly or persistently takes part in firing it, some growth process or metabolic change takes place in one or both cells such that A’s efficiency, as one of the cells firing B, is increased” (Hebb, 1949). Spike timing-dependent plasticity (STDP) is a Hebbian learning rule (Caporale and Dan, 2008; Markram et al., 2011; Feldman, 2012) that is thought to underlie circuit remodeling during development (Feldman and Brecht, 2005; Caporale and Dan, 2008). In STDP, the precise temporal order of spiking in pre- and postsynaptic neurons determines the direction of synaptic modification (potentiation or depression) (Markram et al., 1997; Bi and Poo, 1998; Debanne et al., 1998; Feldman, 2000). However, it is unclear to what extent natural activity patterns engage STDP or other mechanisms to alter synaptic weights during development (Paulsen and Sejnowski, 2000; Froemke and Dan, 2002). In order to identify relevant spike patterns during cortical development, we recorded neuronal spiking activity in developing mouse barrel cortex in response to sensory stimulation. Specifically, we were interested in the spike patterns of layer 4 cells during the third postnatal week, a critical period of refinement of their synaptic connections onto layer 2/3 cells (Fox et al., 1996; Wen and Barth, 2011). We found that these activity patterns, replayed as presynaptic input onto layer 2/3 cells, were sufficient to drive synaptic long-term depression (LTD). Surprisingly, similar spike patterns replayed in individual presynaptic layer 4 cells induced LTD without a requirement for postsynaptic or astroglial signaling. This presynaptic spike pattern-dependent form of LTD may complement timing-dependent LTD as a developmental learning rule balancing Hebbian potentiation.

## Results

**Figure 1:**
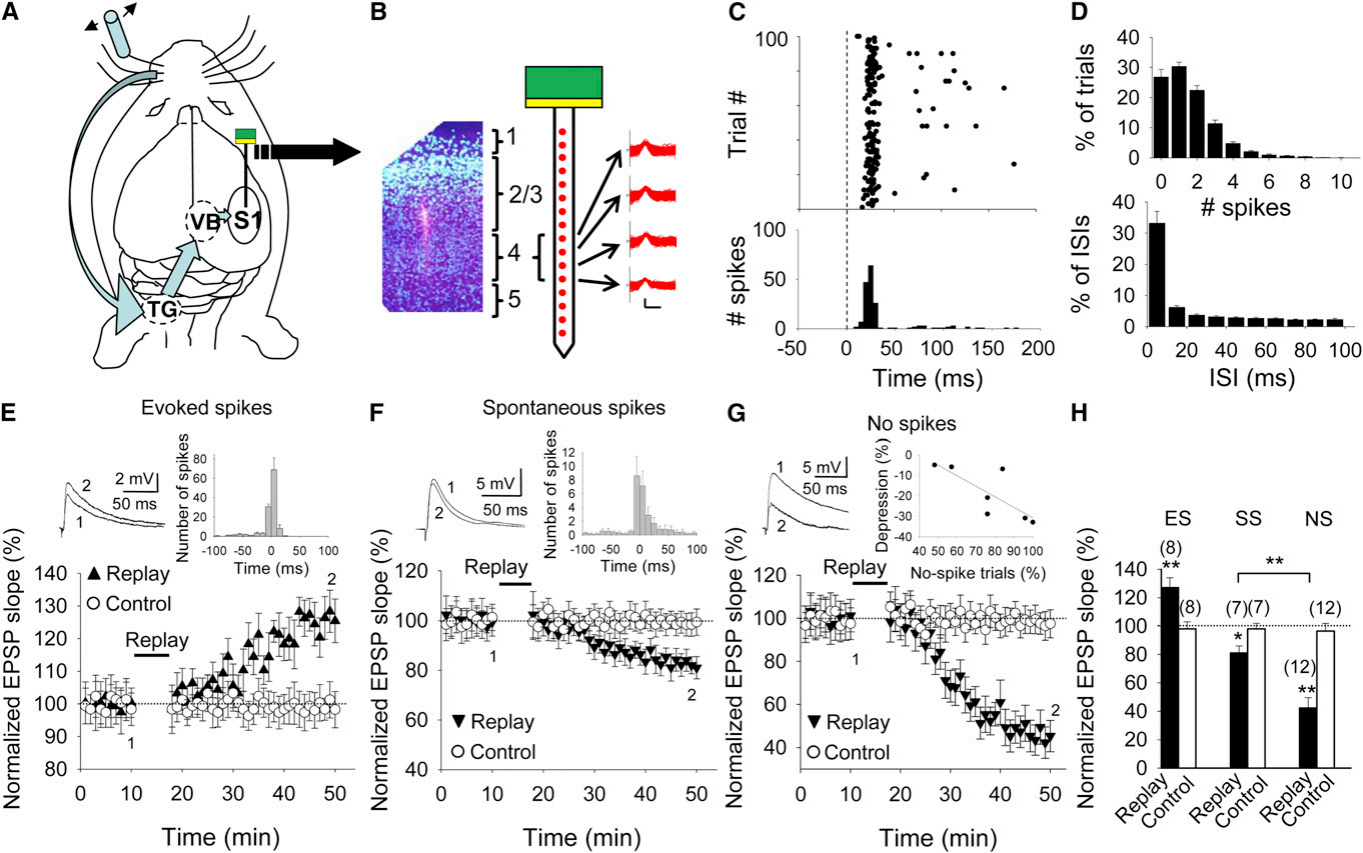
Replay of In Vivo Presynaptic Activity Induces Synaptic Plasticity at Layer 4 to Layer 2/3 Synapses (A) Schematic showing the path of neural signals from stimulation of whisker via the trigeminal nucleus (TG), ventrobasal thalamus (VB), and primary somatosensory cortex (S1). (B) Recordings were made with a linear array of 16 electrodes. Left: coronal section through S1 with neuronal nuclei stained with DAPI (blue) and the DiI-labeled track made by the recording electrode (red). Right: spikes recorded at each of the four electrodes sampling layer 4. (C) Top: raster plot of 100 recording trials of layer 4 unit in response to whisker deflection (time 0). Each black dot represents a spike. Bottom: spike-time histogram. (D) Top: histogram of spike number (mean ± SEM) recorded within 200 ms after whisker deflection in 20 units. Bottom: histogram of interspike intervals (ISIs) in the same units. (E–G) Replay of presynaptic spike trains obtained in vivo induces synaptic potentiation when presynaptic stimulation evokes postsynaptic spikes (E), synaptic depression in spontaneously firing neurons (F), and prominent depression in nonspiking neurons (G). Inset traces show the EPSP before (1) and 30 min after (2) the replay protocol. Insets in (E) and (F) show the distribution of time differences between presynaptic stimulus and postsynaptic spikes during the replay period. Bin, 10 ms. Inset in (G) shows the amount of depression against proportion of trials without spikes. (H) Summary of results. Error bars indicate SEM. ^∗∗^p < 0.01, ^∗^p < 0.05, Student’s t test. The number of slices for each protocol is indicated in parentheses at the top of each error bar. See also Table S1.

Extracellular recordings were made from 20 single units in layer 4 of barrel cortex from five 18-day-old mice. In response to whisker deflections (Figures 1A and 1B), these units typically produced a brief burst of spikes followed by occasional single spikes over the next 200 ms (Figure 1C). The number of spikes evoked by whisker deflection varied between trials. Of those cells that responded within 200 ms to sensory input with spikes in at least some trials, no spikes were detected in 27% ± 12% of trials, and more than three spikes were detected in 9% ± 5% of trials (mean ± SD; n = 20; Figure 1D, top). Cells shared a strong tendency for bursting activity as, within the first 50 ms after stimulus onset, 14% ± 8% of trials showed a burst of three or more action potentials, with an average of 1.23 ± 0.42 spikes/trial in this period. In addition, within 200 ms after stimulation, almost 40% of the interspike intervals were less than 20 ms (39% ± 16%, mean ± SD; n = 20; Figure 1D, bottom).

To investigate the effect of such sensory-evoked spike trains on synaptic efficacy, we used the spike trains recorded from layer 4 cells in 100 consecutive trials to stimulate layer 4 input onto layer 2/3 cells in a slice preparation from the barrel cortex at a similar developmental age (10–21 postnatal days). In this age range, this synapse expresses STDP (Feldman, 2000; Banerjee et al., 2009). First, we allowed the postsynaptic neuron to fire action potentials in response to this afferent input by adjusting the stimulus strength of afferent input so that postsynaptic spikes were elicited on almost every trial. Most of the spikes evoked in the postsynaptic cell appeared within 10 ms after the presynaptic input (Figure 1E, inset) and, as predicted from Hebb’s learning rule (pre-before-post pairing), synaptic potentiation was induced (127% ± 7% versus 98% ± 5% in the control pathway, n = 8; p < 0.01, paired t test; Figure 1E). Second, we depolarized the postsynaptic neuron so that it spontaneously fired spikes. Replaying the same synaptic input during this condition led to LTD (81% ± 5% versus 98% ± 4% in the control pathway, n = 7; p < 0.05, paired t test; Figure 1F). This is an expected outcome, because the time window for induction of depression by post-before-pre pairing is much wider than the time window for induction of potentiation by pre-before-post pairing (Feldman, 2000). Thus, spontaneous firing in postsynaptic neurons would induce net synaptic depression. To our surprise, however, we noticed that the magnitude of depression was greater when there were fewer trials containing postsynaptic spikes (Figure 1G, inset). We therefore asked whether postsynaptic spikes are necessary at all for the induction of this form of LTD. To test this, we prevented the postsynaptic neuron from firing by injecting hyperpolarizing current (50–120 pA) during replay of presynaptic activity. We found that this led to an even bigger depression (42% ± 7% versus 96% ± 5% in the control pathway, n = 12; p < 0.01, paired t test; Figure 1G). Overall, replay of the same presynaptic spike train led to potentiation when the postsynaptic cell was allowed to fire spikes in response to synaptic input but to depression when the postsynaptic cell was spontaneously active (Figure 1H). Unexpectedly, though, the biggest depression was seen during replay of presynaptic input when the postsynaptic cell was silent (p < 0.01, t test NS versus SS; Figure 1H).

**Figure 2:**
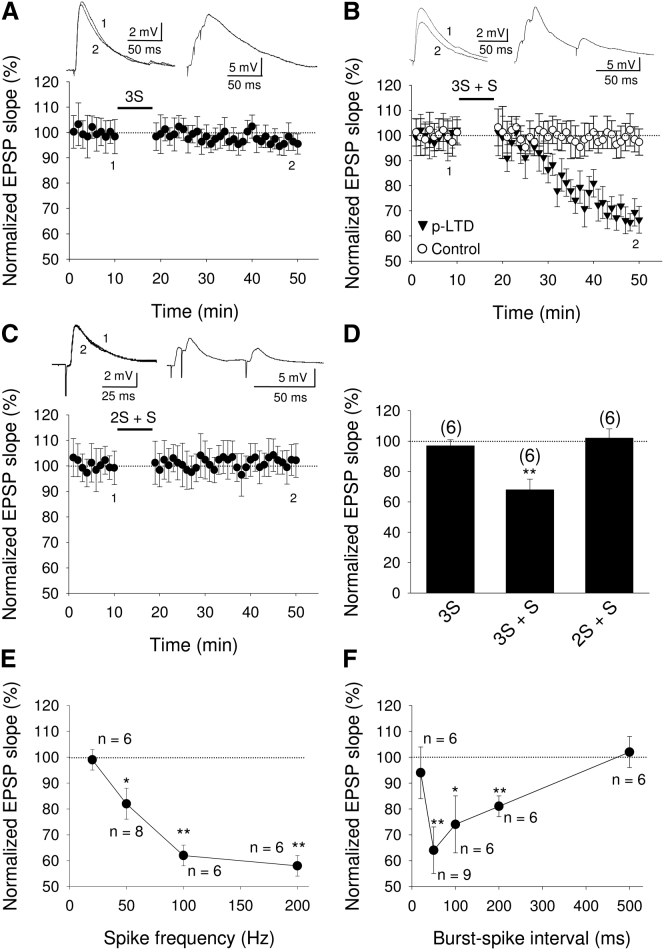
Presynaptic Burst of Three Spikes Followed by Single Spike Induces LTD (A) Burst of three spikes (3S) fails to induce LTD. (B) Presynaptic burst of three spikes followed by single presynaptic spike (3S + S) induces LTD. (C) Burst of two spikes plus a single spike (2S + S) does not induce LTD. Insets show effect of protocol on EPSP (left) and the membrane potential response during the protocol (right). (D) Summary of results. The number of slices for each protocol is indicated at the top of each error bar. (E and F) Effect of different spike frequency in the burst (E) and time from last spike in burst to presynaptic single spike (F) on the magnitude of synaptic depression. Error bars indicate SEM. ^∗∗^p < 0.01, ^∗^p < 0.05, Student’s t test. See also Figure S1.

What features of the presynaptic spike train enable the induction of LTD in the absence of postsynaptic spikes? Since presynaptic bursting activity was often seen in response to sensory stimulation (Figures 1C and 1D), we first tested whether a presynaptic burst of three spikes at 100 Hz was sufficient to induce LTD when the postsynaptic neuron was silenced by hyperpolarizing current (50–120 pA). However, no change in synaptic efficacy was observed after 100 presynaptic bursts of activity (95% ± 3% versus 97% ± 4% in the control pathway, n = 6; Figure 2A). Since many layer 4 unit bursts in vivo were followed by occasional single spikes within a 200 ms time window, we next tried to mimic such spike trains by adding a single presynaptic stimulation 50 ms after the presynaptic burst of three spikes. This stimulation paradigm led to robust input-specific LTD (68% ± 7% versus 99% ± 6% in the control pathway, n = 6; p < 0.01, paired t test; Figure 2B; see Figure S1 available online). In contrast, a presynaptic burst comprising only two spikes followed by a single presynaptic spike did not induce LTD (102% ± 6% versus 107% ± 8% in the control pathway, n = 6; Figure 2C). Therefore, we conclude that a minimum requirement for induction of this form of LTD is a burst of three presynaptic spikes followed by a single presynaptic spike (Figure 2D). To further explore the induction requirements for this presynaptic spike pattern-dependent LTD (p-LTD), we investigated the effect of spike frequency within the burst. We found that a 20 Hz burst did not induce p-LTD (99% ± 4%, n = 6 versus 101% ± 9% in the control pathway), but p-LTD was induced with 50 Hz (82% ± 6%, n = 8 versus 94% ± 8% in control pathway), 100 Hz (62% ± 4%, n = 6 versus 101% ± 5% in the control pathway), or 200 Hz burst (58% ± 4%, n = 6 versus 96% ± 7% in the control pathway) (Figure 2E). Next, we investigated the time window in which the presynaptic single spike must occur in order to induce p-LTD. We found that a 20 ms delay between the presynaptic burst and the presynaptic single spike was not sufficient but that a 50 ms, 100 ms, or 200 ms time interval led to significant depression (Figure 2F). However, no depression was observed when the delay was extended to 500 ms (Figure 2F). We conclude that a burst of three presynaptic spikes at a frequency of at least 50 Hz followed, within a time window of 50–200 ms, by a single presynaptic spike can induce p-LTD.

**Figure 3:**
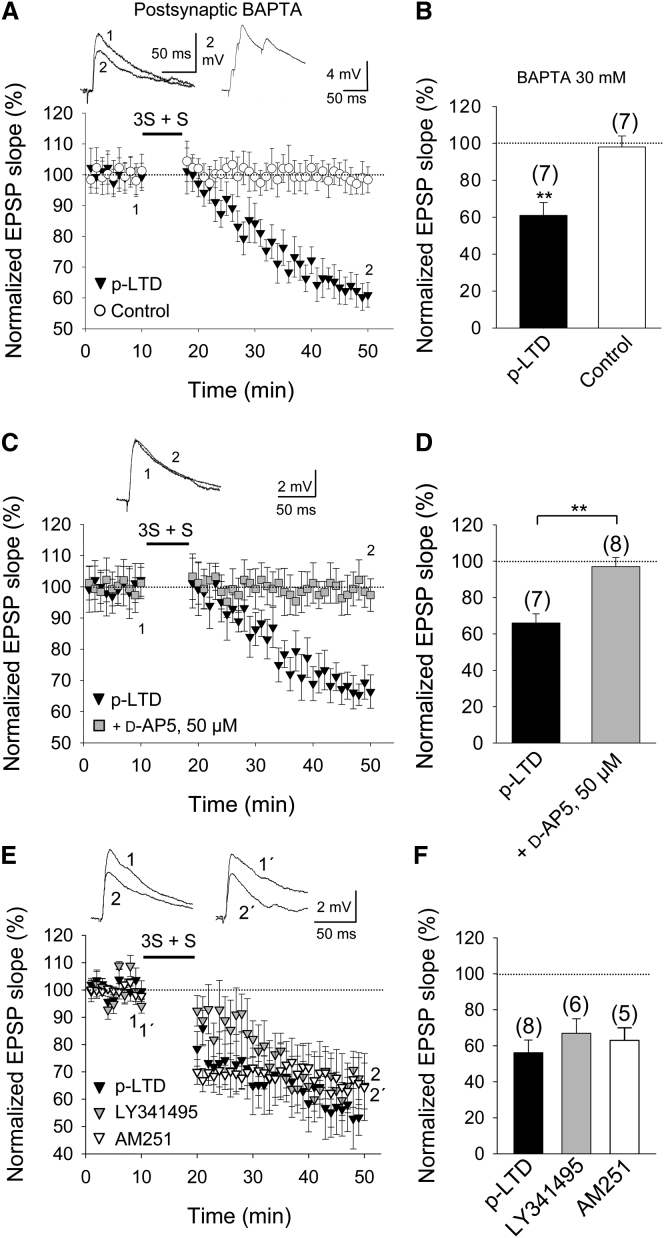
Properties of p-LTD (A) Input-specific p-LTD with BAPTA (30 mM) in the postsynaptic recording pipette. Insets show EPSP before (1) and after (2) p-LTD (left) and the membrane potential response during the protocol (right). (B) Summary of results. (C) p-LTD requires NMDA receptors. p-LTD is completely blocked in 50 μM d-AP5 (gray squares). (D) Summary of results. (E) p-LTD does not require activation of mGlu or CB1 receptors. p-LTD induced by a 3S + S protocol in control slices (black triangles) and in slices treated with the mGluR antagonist LY341495 (100 μM; gray triangles) or the CB1 receptor antagonist AM251 (3 μM; open triangles) is shown. Insets show EPSP before (1 and 1′) and 30 min after (2 and 2′) p-LTD induction protocol in LY341495-treated slices (1 and 2) and in AM251-treated slices (1′ and 2′). (F) Summary of results. The number of slices used for each condition is indicated in parentheses at the top of each bar. Error bars represent SEM. ^∗∗^p < 0.01, Student’s t test. See also Figures S2 and S3.

To compare and contrast p-LTD with timing-dependent LTD (t-LTD), we investigated the cellular mechanisms involved. Induction of p-LTD did not require postsynaptic Ca^2+^, as robust input-specific p-LTD was seen after loading of the Ca^2+^ chelator BAPTA (30 mM) into the postsynaptic neuron (61% ± 7% versus control 98% ± 6%, n = 7; p < 0.01, paired t test; Figures 3A and 3B), and therefore all subsequent experiments on p-LTD were performed with BAPTA in the postsynaptic recording pipette. Similar to t-LTD (Feldman, 2000), the induction of p-LTD requires NMDA receptors, as synaptic depression was not seen with this protocol in the presence of the NMDA receptor antagonist d-2-amino-5-phosphonopentanoic acid (d-AP5, 50 μM) (97% ± 5% versus interleaved control p-LTD 66% ± 5%, n = 7; p < 0.01, t test; Figures 3C and 3D). In contrast, whereas t-LTD also requires metabotropic glutamate receptors (mGluRs), CB1 receptors, and release of glutamate from astrocytes (Sjöström et al., 2003; Bender et al., 2006; Nevian and Sakmann, 2006; Min and Nevian, 2012; but see Hardingham et al., 2008; Banerjee et al., 2009), neither the mGluR antagonist LY341495 (100 μM) nor the CB1 receptor antagonist AM251 (3 μM) affected the induction of p-LTD (67% ± 8%, n = 6 and 63% ± 7%, n = 5, respectively; Figures 3E and 3F). Moreover, an NMDA receptor-dependent form of p-LTD was still induced after treatment with the gliotoxin fluoroacetate (10 mM); in contrast, we found that t-LTD was completely blocked (Figure S2). We conclude that, similar to t-LTD, p-LTD requires NMDA receptors but, in contrast to t-LTD, p-LTD does not require mGluRs, postsynaptic Ca^2+^-dependent processes, CB1 receptors, or astrocytic metabolism.

What is the origin of glutamate that activates these NMDA receptors? For t-LTD, it was recently proposed that astrocytes serve as the source of glutamate (Min and Nevian, 2012). For p-LTD, one possibility is that glutamate released during the burst of three presynaptic spikes preceding the single presynaptic spike is responsible. To test this idea, we investigated whether exogenous glutamate application could substitute for the presynaptic burst. We found that the pairing of local uncaging of glutamate (from MNI-caged glutamate; 100 μM; see Supplemental Experimental Procedures) and a single presynaptic stimulation 50 ms later, repeated 30 times, induced LTD (80% ± 4%, n = 8; p < 0.05, t test). This depression was NMDA receptor dependent, as LTD was not seen in the presence of 50 μM d-AP5 (99% ± 3%, n = 5; p < 0.05, t test against glutamate-induced LTD; Figure S3). Thus, the pairing of glutamate release followed by a single presynaptic spike is sufficient to induce LTD, bypassing the requirement of glutamate release from either the presynaptic neuron (p-LTD) or astrocytes (t-LTD).

**Figure 4:**
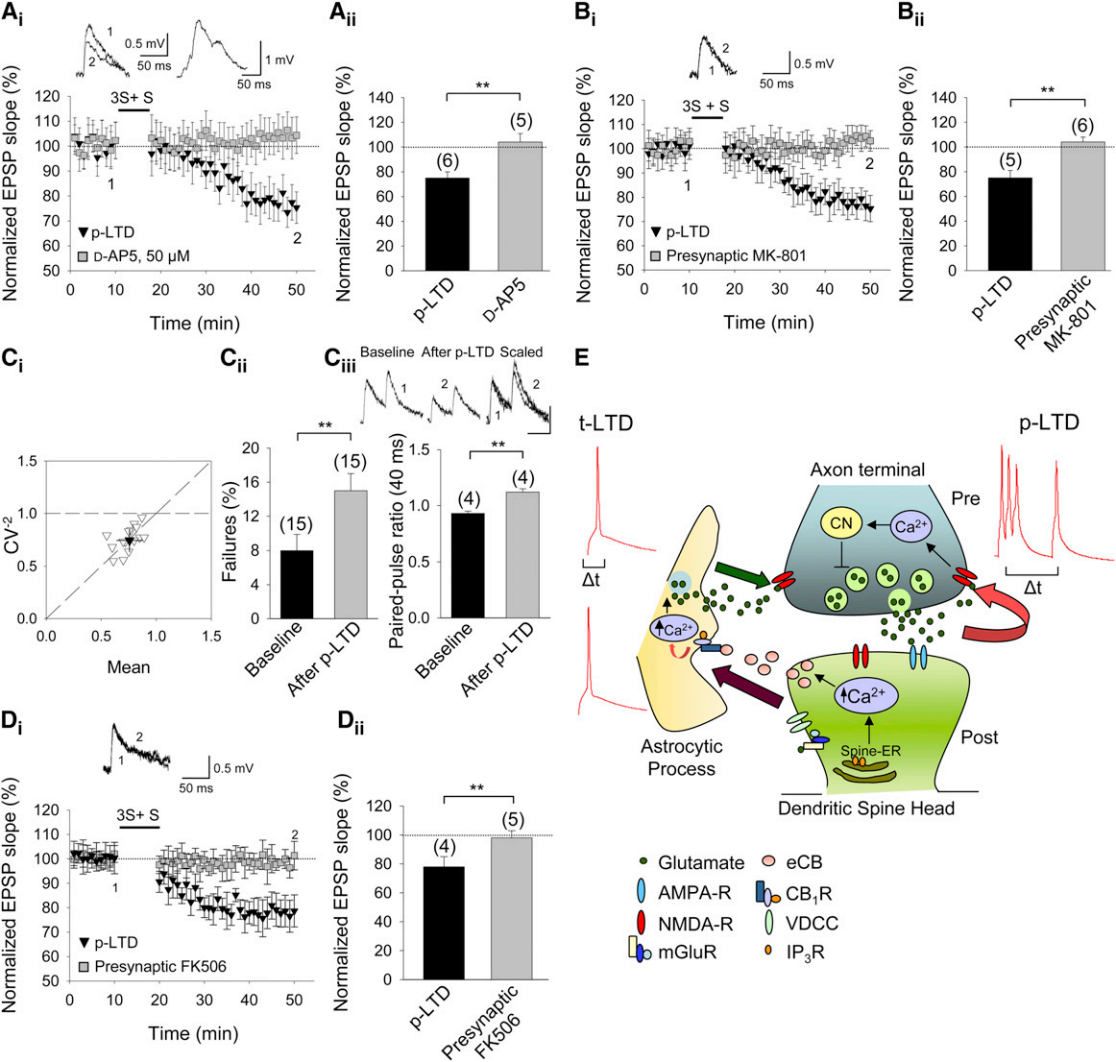
Presynaptic Self-Depression in Individual Presynaptic Layer 4 Neurons (Ai–Bii) Presynaptic NMDA receptor-dependent p-LTD in pairs of synaptically connected layer 4 to layer 2/3 neurons. (Ai) EPSP slopes monitored in d-AP5-treated (gray squares) and nontreated (black triangles) cells with BAPTA in the postsynaptic pipette. Insets show the effect of the protocol on EPSP (left) and the membrane potential response during the protocol (right). (Aii) Summary of results. (Bi) MK801 in the presynaptic pipette blocked the induction of p-LTD. The postsynaptic pipette contained both BAPTA and CsF. Symbols and traces are as in (Ai). (Bii) Summary of results. (Ci–Ciii) p-LTD expression is presynaptic. (Ci) Normalized plot of CV^−2^ versus mean EPSP slope yields data points along the diagonal after induction of p-LTD. Mean of all cells is shown with black triangle. (Cii) Number of failures increases after p-LTD induction. (Ciii) Paired-pulse ratio increases after p-LTD. Example traces during baseline (1) and 30 min after induction of p-LTD (2) are shown. Scale bars represent 50 ms and 0.5 mV. (Di and Dii) p-LTD requires presynaptic calcineurin. (Di) FK506 in the presynaptic pipette blocked induction of p-LTD. Symbols and traces are as in (Ai). (Dii) Summary of results. Error bars represent SEM. ^∗∗^p < 0.01, Student’s t test. The number of slices used for each condition is indicated in parentheses at the top of each error bar. (E) Two forms of presynaptic NMDA receptor-dependent LTD at L4–L2/3 synapses. Left: t-LTD during post-before-pre pairing, postsynaptic action potentials activate voltage-dependent calcium channels (VDCCs), and presynaptically released glutamate activates postsynaptic mGluRs, which synergistically activate PLC, leading to the postsynaptic generation and release of endocannabinoid (eCB). The eCB signal leads to activation of astroglial CB1 receptors, which facilitates glutamate release from astrocytes, activating presynaptic NMDA receptors in layer 4 cells. Right: p-LTD requires neither mGlu nor CB1 receptors. A burst of three presynaptic action potentials evokes glutamate release that activates presynaptic NMDA receptors when followed by a single presynaptic spike. This leads to an increase in presynaptic calcium and synaptic depression, requiring calcineurin (CN), without the involvement of the postsynaptic neuron or astrocytes. See also Figure S4.

To investigate whether individual presynaptic layer 4 neurons could drive their own synaptic depression using a p-LTD protocol, we performed paired whole-cell recordings of synaptically connected layer 4 and layer 2/3 cells. Out of 315 pairs recorded, 40 pairs showed a monosynaptic excitatory postsynaptic potential (EPSP), 39 of which were used in plasticity experiments. In six pairs, the p-LTD protocol was applied to the presynaptic neuron in the presence of 30 mM BAPTA in the postsynaptic pipette. Robust p-LTD was induced (75% ± 5%, n = 6; p < 0.01, t test; Figures 4Ai and 4Aii). Even when all Ca^2+^- and G protein-dependent mechanisms in the postsynaptic neuron were blocked by recording with 30 mM BAPTA and 120 mM CsF in the postsynaptic pipette in five pairs, robust p-LTD was still induced (75% ± 6%, n = 5, p < 0.01, t test; Figures 4Bi and 4Bii). The independence of p-LTD from postsynaptic Ca^2+^- and G protein-dependent mechanisms is in contrast to postsynaptic depolarization-induced, endocannabinoid-dependent presynaptic LTD in layer 5 neurons (Sjöström et al., 2004). To corroborate that this p-LTD requires NMDA receptors, we recorded five pairs in the presence of 50 μM d-AP5. In this condition, the induction of p-LTD was completely blocked (104% ± 7%, n = 5; p < 0.01, t test against control p-LTD; Figures 4Ai and 4Aii). To determine the location of these NMDA receptors, we repeated the experiment with the presynaptic cell loaded with the NMDA receptor channel blocker MK-801 (Figures 4Bi and 4Bii). In six pairs with the presynaptic cell loaded with 1 mM MK801, p-LTD was completely blocked (103% ± 4%, n = 6; p < 0.01, t test against control p-LTD). Thus, similar to t-LTD (Rodríguez-Moreno and Paulsen, 2008; Rodríguez-Moreno et al., 2011), presynaptic NMDA receptors are required for p-LTD. If these forms of LTD converge mechanistically on presynaptic NMDA receptors, they should occlude each other. Indeed, we found that the p-LTD protocol failed to induce further depression after t-LTD had been induced (Figure S4A) and, conversely, that the t-LTD protocol failed to induce further depression after p-LTD had been induced (Figure S4B). Moreover, fluctuation analysis (Figure 4Ci), failure analysis (Figure 4Cii), and analysis of paired-pulse ratios (PPRs) from the paired recordings (Figure 4Ciii) confirmed that the locus of expression of p-LTD is also presynaptic.

Finally, to gain mechanistic insight into how activation of presynaptic NMDA receptors can lead to presynaptic LTD, we conjectured that a presynaptic Ca^2+^-dependent enzyme might be involved. Since the Ca^2+^-dependent protein phosphatase calcineurin has earlier been implicated in LTD, albeit in the postsynaptic neuron, both in the hippocampus (Mulkey et al., 1994) and neocortex (Torii et al., 1995), we tested the effect of the calcineurin inhibitor FK506. p-LTD was completely blocked by either extracellular application of FK506 (10 μM; data not shown) or presynaptic intracellular delivery of FK506 (10 μM; 98% ± 5%, n = 5 versus 78% ± 7%, n = 4 in interleaved slices; p < 0.01, t test; Figures 4Di and 4Dii). Together, these results show that natural spike patterns in layer 4 neurons can induce presynaptic calcineurin-dependent LTD at layer 4 to layer 2/3 synapses by the activation of presynaptic NMDA receptors on individual presynaptic neurons independent of postsynaptic and astroglial signaling (Figure 4E).

## Discussion

In this study, we examined the effect of presynaptic spike trains recorded from cortical layer 4 neurons in vivo on the strength of layer 4 to layer 2/3 synapses in vitro. We found that a specific spike pattern displayed by layer 4 neurons in vivo, consisting of a brief burst of spikes (≥50 Hz) followed within a restricted time window (50–200 ms) by a single spike, induces LTD at excitatory synapses on postsynaptically silent layer 2/3 neurons. This presynaptic spike pattern-dependent form of LTD can be induced by individual presynaptic layer 4 cells and requires presynaptic NMDA receptors and calcineurin but, in contrast to timing-dependent LTD, is independent of postsynaptic and astrocytic signaling.

Presynaptic NMDA receptors (Duguid and Sjöström, 2006; Corlew et al., 2008; Buchanan et al., 2012) are required for both t-LTD and p-LTD. However, the source of glutamate activating these receptors appears to differ. Recent evidence suggests that t-LTD is mediated by astroglial release of glutamate acting on presynaptic NMDA receptors (Min and Nevian, 2012). However, the immediate post-pre pairing detector appears to be postsynaptic, involving mGluRs and retrograde endocannabinoid signaling acting on astroglial CB1 receptors at developing layer 4 to layer 2/3 synapses (Bender et al., 2006; Min and Nevian, 2012; Figure S2, left). A similar mechanism is likely for the postsynaptic depolarization-induced, endocannabinoid-dependent presynaptic LTD that has been reported in visual cortical layer 5 neurons (Sjöström et al., 2004). By contrast, we found no evidence that electrical or metabolic processes in the postsynaptic neuron or astroglia are required for p-LTD, and the most parsimonious explanation is that the source of glutamate responsible for the activation of presynaptic NMDA receptors in p-LTD is the presynaptic neuron itself. Indeed, the burst of presynaptic activity required for p-LTD could be replaced by exogenous glutamate application (Figure S3). Given that p-LTD and t-LTD mutually occlude each other (Figure S4), an attractive possibility is that the post-pre pairing in t-LTD and the presynaptic burst in p-LTD both trigger the release of glutamate, from astroglia or axon terminals, respectively, and mechanistically converge on presynaptic NMDA receptors. These mechanisms are not mutually exclusive and might work in concert, with glutamate released from both presynaptic boutons and astroglia. Since both t-LTD and p-LTD require presynaptic activity in addition to glutamate release (Min and Nevian, 2012; this study), it is possible that the presynaptic LTD mechanism in both cases utilizes the coincidence detector properties of NMDA receptors engendered by their voltage-dependent Mg^2+^ block (Mayer et al., 1984; Nowak et al., 1984) but that the primary coincidence detector for t-LTD is postsynaptic, offering a possible explanation for the different time windows for induction of t-LTD and p-LTD. The voltage-dependent properties of NMDA receptors depend on their subunit composition (see Paoletti, 2011), and both Mg^2+^-sensitive and -insensitive presynaptic NMDA receptors have been reported (McGuinness et al., 2010; Larsen et al., 2011). GluN2A subunit-containing presynaptic NMDA receptors have been implicated in cerebellar LTD (Bidoret et al., 2009). While the broad time window for induction of p-LTD in the barrel cortex would be consistent with the expression of slow GluN2C/D-containing NMDA receptors in layer 4 cells (Binshtok et al., 2006; Banerjee et al., 2009), the precise subunit composition of presynaptic NMDA receptors in layer 4 neurons in the mouse barrel cortex remains unknown, and it is possible that other factors are involved in the induction of p-LTD.

What are the functional implications of p-LTD? Hebb’s neurophysiological postulate suggests a rule for synaptic potentiation, by which the connection from cell A to cell B strengthens when cell A “repeatedly or persistently takes part in firing” cell B (Hebb, 1949). Spike timing-based rules suggest that synaptic depression should occur if the spike order is reversed, such that cell A does not take part in firing cell B, and there is strong experimental evidence that cortical synapses express such t-LTD (see Caporale and Dan, 2008). Spike pattern-based rules may complement timing-based rules, offering two mechanisms to compete with Hebbian potentiation: t-LTD occurs when the presynaptic neuron fires too late to take part in firing the postsynaptic neuron, and p-LTD occurs when vigorous activity in a presynaptic neuron fails to activate the postsynaptic neuron, in line with theoretical predictions (Stent, 1973; Lisman, 1989). This latter learning rule could potentially contribute to postsynaptic action potential-independent systems-level plasticity (Reiter and Stryker, 1988). Similar to t-LTD (Banerjee et al., 2009), p-LTD was found only until 3 weeks of age (Figure S1), suggesting that it might be a mechanism for synaptic depression preceding elimination of inappropriate synapses during development (Kamikubo et al., 2006; Bastrikova et al., 2008; Holtmaat and Svoboda, 2009). These complementary learning rules add to the computational repertoire of developing cortical networks and both are likely to contribute to the changes in synaptic efficacy that shape functional circuit architecture during development.

## Experimental Procedures

### Animal Experiments

All animal procedures were in accordance with UK Animals (Scientific Procedures) Act 1986. C57BL6 mouse pups were anesthetized with isofluorane and placed in a stereotaxic frame or decapitated for slice preparation. Unit firing was recorded with a single-shank silicon probe electrode with 16 sites vertically aligned at 50 μm intervals (see Table S1). Voltage recordings were band-pass filtered (500 Hz–5 kHz), amplified, and digitized at 25 kHz. Whisker stimulation was achieved with a piezoelectric wafer.

### Acute Slice Experiments

Slices from the barrel cortex were maintained at room temperature (22°C–27°C) in artificial cerebrospinal fluid containing 126 mM NaCl, 3 mM KCl, 1.25 mM NaH_2_PO_4_, 2 mM MgSO_4_, 2 mM CaCl_2_, 26 mM NaHCO_3_, and 10 mM glucose (pH 7.2–7.4), bubbled with carbogen gas (95% O_2_/5% CO_2_). Two monopolar stimulation electrodes were placed at the base of a barrel (layer 4). Whole-cell voltage recordings were obtained from layer 2/3 pyramidal cells in the same barrel column with 5–7 MΩ borosilicate pipettes containing 110 mM potassium gluconate, 40 mM HEPES, 4 mM NaCl, 4 mM ATP-Mg, and 0.3 mM GTP, adjusted to pH 7.2 with KOH. Recordings were low-pass filtered at 2 kHz and acquired at 5 kHz.

EPSPs were evoked alternately in two input pathways, test and control, each at 0.2 Hz, or, in paired recording, by a single presynaptic layer 4 neuron. After a stable EPSP baseline period of at least 10 min, a plasticity protocol was applied to the test pathway. The EPSPs were monitored for 30 min after the end of the plasticity protocol. Plasticity was assessed from the slope of the EPSP, measured on the rising phase of the EPSP as a linear fit between time points corresponding to 25%–30% and 70%–75% of the peak amplitude during control conditions.

Drugs were purchased from Tocris Bioscience and Sigma.

Statistical comparisons were made using one-sample, two-sample, or paired Student’s t test as appropriate. p values less than 0.05 were considered significant. Data are presented as mean ± SEM unless otherwise indicated.

Further experimental details are available in the Supplemental Experimental Procedures.

## Supplemental Information

Document S1. Figures S1–S4, Table S1, and Supplemental Experimental Procedures

## References

[bib1] Banerjee A., Meredith R.M., Rodríguez-Moreno A., Mierau S.B., Auberson Y.P., Paulsen O. (2009). Double dissociation of spike timing-dependent potentiation and depression by subunit-preferring NMDA receptor antagonists in mouse barrel cortex. Cereb. Cortex.

[bib2] Bastrikova N., Gardner G.A., Reece J.M., Jeromin A., Dudek S.M. (2008). Synapse elimination accompanies functional plasticity in hippocampal neurons. Proc. Natl. Acad. Sci. USA.

[bib3] Bender V.A., Bender K.J., Brasier D.J., Feldman D.E. (2006). Two coincidence detectors for spike timing-dependent plasticity in somatosensory cortex. J. Neurosci..

[bib4] Bi G.-Q., Poo M.M. (1998). Synaptic modifications in cultured hippocampal neurons: dependence on spike timing, synaptic strength, and postsynaptic cell type. J. Neurosci..

[bib5] Bidoret C., Ayon A., Barbour B., Casado M. (2009). Presynaptic NR2A-containing NMDA receptors implement a high-pass filter synaptic plasticity rule. Proc. Natl. Acad. Sci. USA.

[bib6] Binshtok A.M., Fleidervish I.A., Sprengel R., Gutnick M.J. (2006). NMDA receptors in layer 4 spiny stellate cells of the mouse barrel cortex contain the NR2C subunit. J. Neurosci..

[bib7] Buchanan K.A., Blackman A.V., Moreau A.W., Elgar D., Costa R.P., Lalanne T., Tudor Jones A.A., Oyrer J., Sjöström P.J. (2012). Target-specific expression of presynaptic NMDA receptors in neocortical microcircuits. Neuron.

[bib8] Caporale N., Dan Y. (2008). Spike timing-dependent plasticity: a Hebbian learning rule. Annu. Rev. Neurosci..

[bib9] Corlew R., Brasier D.J., Feldman D.E., Philpot B.D. (2008). Presynaptic NMDA receptors: newly appreciated roles in cortical synaptic function and plasticity. Neuroscientist.

[bib10] Debanne D., Gähwiler B.H., Thompson S.M. (1998). Long-term synaptic plasticity between pairs of individual CA3 pyramidal cells in rat hippocampal slice cultures. J. Physiol..

[bib11] Duguid I., Sjöström P.J. (2006). Novel presynaptic mechanisms for coincidence detection in synaptic plasticity. Curr. Opin. Neurobiol..

[bib12] Feldman D.E. (2000). Timing-based LTP and LTD at vertical inputs to layer II/III pyramidal cells in rat barrel cortex. Neuron.

[bib13] Feldman D.E. (2012). The spike-timing dependence of plasticity. Neuron.

[bib14] Feldman D.E., Brecht M. (2005). Map plasticity in somatosensory cortex. Science.

[bib15] Fox K., Glazewski S., Chen C.M., Silva A., Li X. (1996). Mechanisms underlying experience-dependent potentiation and depression of vibrissae responses in barrel cortex. J. Physiol. Paris.

[bib16] Froemke R.C., Dan Y. (2002). Spike-timing-dependent synaptic modification induced by natural spike trains. Nature.

[bib17] Hardingham N., Wright N., Dachtler J., Fox K. (2008). Sensory deprivation unmasks a PKA-dependent synaptic plasticity mechanism that operates in parallel with CaMKII. Neuron.

[bib18] Hebb D.O. (1949). The Organization of Behavior.

[bib19] Holtmaat A., Svoboda K. (2009). Experience-dependent structural synaptic plasticity in the mammalian brain. Nat. Rev. Neurosci..

[bib20] Kamikubo Y., Egashira Y., Tanaka T., Shinoda Y., Tominaga-Yoshino K., Ogura A. (2006). Long-lasting synaptic loss after repeated induction of LTD: independence to the means of LTD induction. Eur. J. Neurosci..

[bib21] Larsen R.S., Corlew R.J., Henson M.A., Roberts A.C., Mishina M., Watanabe M., Lipton S.A., Nakanishi N., Pérez-Otaño I., Weinberg R.J., Philpot B.D. (2011). NR3A-containing NMDARs promote neurotransmitter release and spike timing-dependent plasticity. Nat. Neurosci..

[bib22] Lisman J. (1989). A mechanism for the Hebb and the anti-Hebb processes underlying learning and memory. Proc. Natl. Acad. Sci. USA.

[bib23] Markram H., Lübke J., Frotscher M., Sakmann B. (1997). Regulation of synaptic efficacy by coincidence of postsynaptic APs and EPSPs. Science.

[bib24] Markram H., Gerstner W., Sjöström P.J. (2011). A history of spike-timing-dependent plasticity. Front Synaptic Neurosci.

[bib25] Mayer M.L., Westbrook G.L., Guthrie P.B. (1984). Voltage-dependent block by Mg^2+^ of NMDA responses in spinal cord neurones. Nature.

[bib26] McGuinness L., Taylor C., Taylor R.D., Yau C., Langenhan T., Hart M.L., Christian H., Tynan P.W., Donnelly P., Emptage N.J. (2010). Presynaptic NMDARs in the hippocampus facilitate transmitter release at theta frequency. Neuron.

[bib27] Min R., Nevian T. (2012). Astrocyte signaling controls spike timing-dependent depression at neocortical synapses. Nat. Neurosci..

[bib28] Mulkey R.M., Endo S., Shenolikar S., Malenka R.C. (1994). Involvement of a calcineurin/inhibitor-1 phosphatase cascade in hippocampal long-term depression. Nature.

[bib29] Nevian T., Sakmann B. (2006). Spine Ca^2+^ signaling in spike-timing-dependent plasticity. J. Neurosci..

[bib30] Nowak L., Bregestovski P., Ascher P., Herbet A., Prochiantz A. (1984). Magnesium gates glutamate-activated channels in mouse central neurones. Nature.

[bib31] Paoletti P. (2011). Molecular basis of NMDA receptor functional diversity. Eur. J. Neurosci..

[bib32] Paulsen O., Sejnowski T.J. (2000). Natural patterns of activity and long-term synaptic plasticity. Curr. Opin. Neurobiol..

[bib33] Reiter H.O., Stryker M.P. (1988). Neural plasticity without postsynaptic action potentials: less-active inputs become dominant when kitten visual cortical cells are pharmacologically inhibited. Proc. Natl. Acad. Sci. USA.

[bib34] Rodríguez-Moreno A., Paulsen O. (2008). Spike timing-dependent long-term depression requires presynaptic NMDA receptors. Nat. Neurosci..

[bib35] Rodríguez-Moreno A., Kohl M.M., Reeve J.E., Eaton T.R., Collins H.A., Anderson H.L., Paulsen O. (2011). Presynaptic induction and expression of timing-dependent long-term depression demonstrated by compartment-specific photorelease of a use-dependent NMDA receptor antagonist. J. Neurosci..

[bib36] Sjöström P.J., Turrigiano G.G., Nelson S.B. (2003). Neocortical LTD via coincident activation of presynaptic NMDA and cannabinoid receptors. Neuron.

[bib37] Sjöström P.J., Turrigiano G.G., Nelson S.B. (2004). Endocannabinoid-dependent neocortical layer-5 LTD in the absence of postsynaptic spiking. J. Neurophysiol..

[bib38] Stent G.S. (1973). A physiological mechanism for Hebb’s postulate of learning. Proc. Natl. Acad. Sci. USA.

[bib39] Torii N., Kamishita T., Otsu Y., Tsumoto T. (1995). An inhibitor for calcineurin, FK506, blocks induction of long-term depression in rat visual cortex. Neurosci. Lett..

[bib40] Wen J.A., Barth A.L. (2011). Input-specific critical periods for experience-dependent plasticity in layer 2/3 pyramidal neurons. J. Neurosci..

