# Supplementary material for: Presynaptic Self-Depression at Developing Neocortical Synapses
Source: Neuron. 2013 Jan 9;77(1):35–42. doi: 10.1016/j.neuron.2012.10.035 (PMC3542421; doi:10.1016/j.neuron.2012.10.035)
Supplement: Document S1. Figures S1–S4, Table S1, and Supplemental Experimental Procedures [file mmc1.pdf]

Neuron, Volume *77*

Supplemental Information

Presynaptic Self-Depression

at Developing Neocortical Synapses

Antonio Rodríguez-Moreno, Ana González-Rueda, Abhishek Banerjee, A. Louise Upton, Michael T. Craig, and Ole Paulsen

SUPPLEMENTAL FIGURES

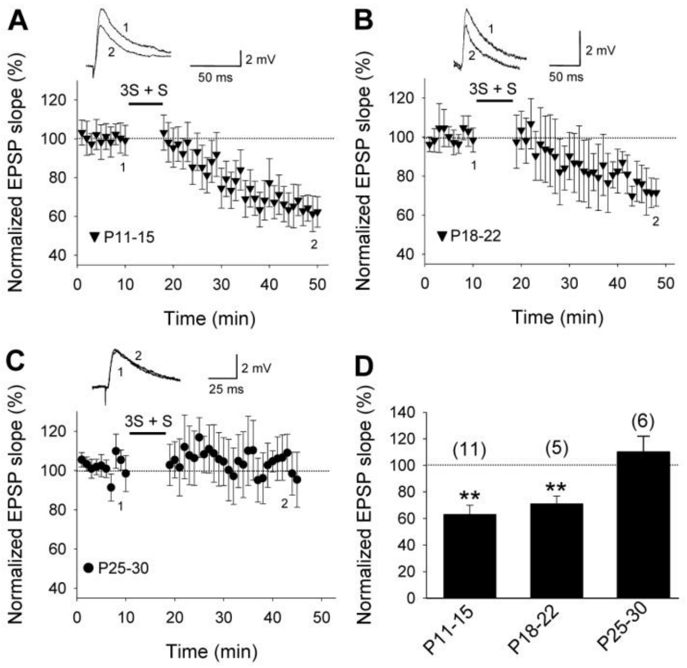

Figure S1, related to Figure 2. Developmental profile of p-LTD at L4-L2/3 synapses in mouse barrel cortex. Synaptic efficacy was monitored over time following p-LTD protocol in (A) P11-15 (B) P18-22 and (C) P25-30 mice. (D) Summary of results. Error bars are SEM. ** $P < 0.01$, Student's *t*-test. The number of slices used for each age group is indicated in parentheses at the top of each error bar.

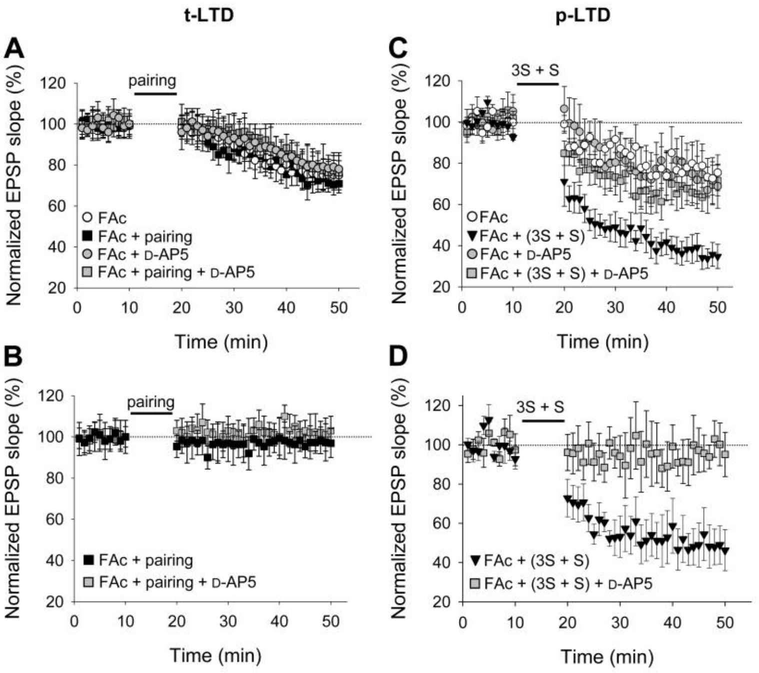

Figure S2, related to Figure 3. Astroglial signalling is required for the induction of t-LTD but not p-LTD. (A) EPSP slope monitored over time in test (black squares) and control pathway (open circles) in fluoroacetate (FAc)-treated slices. A t-LTD protocol (pairing) was applied after 10 minutes to the test pathway. Both test and control pathway show a depression. This depression was not blocked by D-AP5 (gray squares). (B) Difference between test and control pathway plotted over time. No input-specific depression is seen. (C) EPSP slope monitored over time in test (black triangles) and control pathway (open circles) in FAc-treated slices. A p-LTD protocol (3S + S) was applied to the test pathway after 10 minutes in the absence (black triangles) and in the presence of D-AP5 (gray squares). Note that the test pathway shows more depression than the control pathway, and that this depression is prevented by D-AP5. (D) Difference between test and control pathway plotted over time. NMDA receptor-dependent input-specific depression is seen in the test pathway.

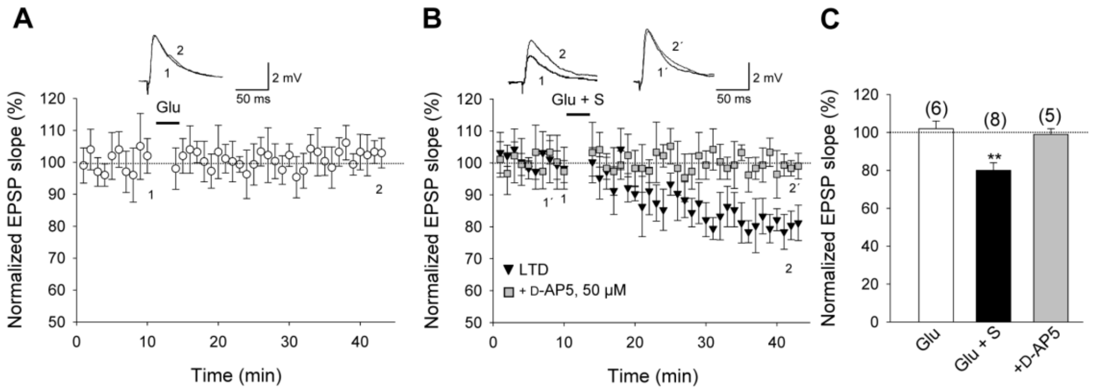

Figure S3, related to Figure 3. Glutamate uncaging followed by a single presynaptic spike 50 ms later induces LTD. (A) Effect of uncaging of MNI-caged glutamate on EPSP slope. Inset traces show the EPSP before (1) and 30 minutes after uncaging of caged glutamate (2). (B) Glutamate uncaging followed 50 ms later by a single presynaptic spike induces LTD (black triangles). In this experiment, postsynaptic cells were loaded with BAPTA (30 mM) via the patch pipette. The induction of LTD requires NMDA receptors. In the presence of D-AP5 the induction of LTD by this protocol was prevented (gray squares). (C) Summary of results. Error bars are SEM. ** $P < 0.01$, Student's *t*-test. The number of slices used for each condition is indicated in parentheses at the top of each error bar.

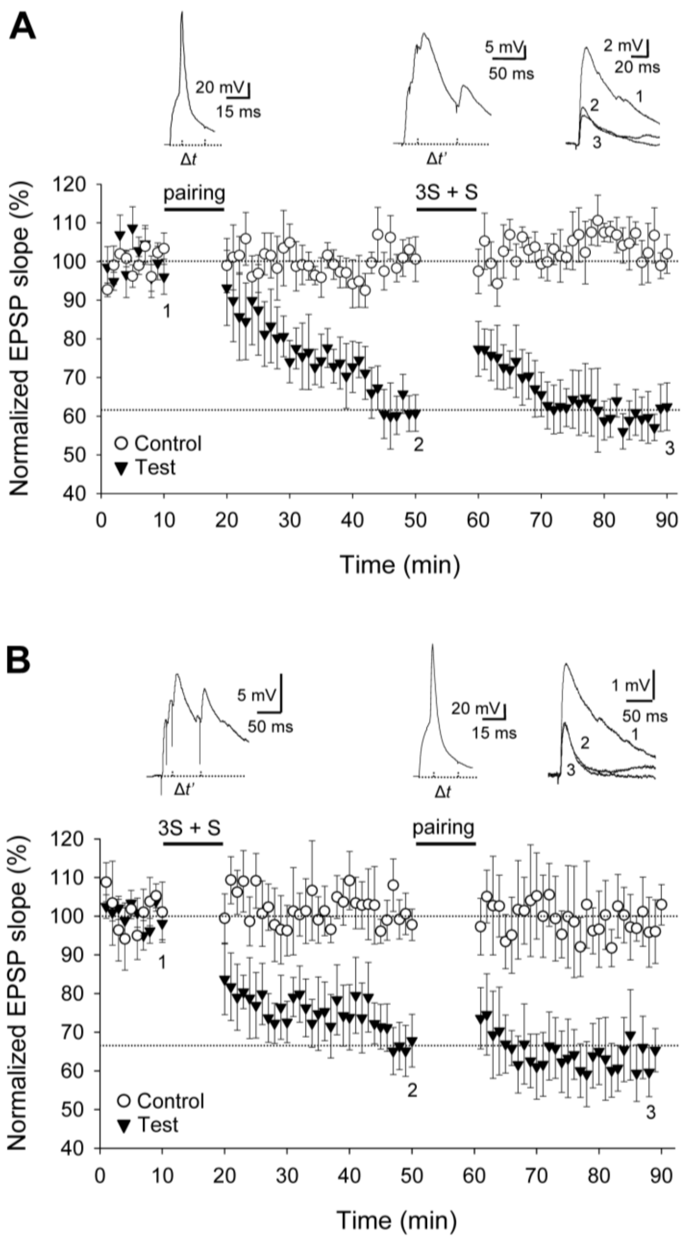

Figure S4, related to Figure 4. Mutual occlusion of t-LTD and p-LTD at L4-L2/3 synapses. (A) EPSP slope monitored over time in a control pathway (open circles) and in neurons undergoing a p-LTD induction protocol (3S + S) after previous induction of t-LTD (black triangles, test pathway). No further depression is induced by the p-LTD protocol (n = 6). Insets show membrane potential response during the protocols (left, post-pre pairing for t-LTD and middle, 3S + S for p-LTD) and the effect of these protocols on EPSP (right). (B) Time course of the effect on EPSP slope of a t-LTD induction protocol (pairing) after the induction of p-LTD. Symbols as in (A). No further depression is induced by a t-LTD induction protocol (n = 6). Insets show membrane potential response during the protocols (left, 3S + S for p-LTD and middle, post-pre pairing for t-LTD) and the effect of these protocols on EPSP (right).

Table S1, related to Figure 1.

Table of the angle of electrode penetration and the electrode channels calculated to be in layer 4. For each recording, the electrode depth corresponding to each cortical layer was calculated based on the histology and the angle of penetration seen in the post-mortem sections.

Mouse ID #	Angle of electrode track	Electrode channels located in layer 4
Mouse #1	88°	9, 10, 11, 12
Mouse #2	66°	10,11,12,13
Mouse #3	72°	9, 10, 11, 12,13
Mouse #4	89°	9, 10, 11, 12
Mouse #5	90°	9, 10, 11, 12

SUPPLEMENTAL EXPERIMENTAL PROCEDURES

In vivo recordings

All animal procedures were in accordance with UK Animals (Scientific Procedures) Act 1986. Five 18-day old C57BL6 mouse pups were anesthetized with isofluorane delivered in oxygen (concentration adjusted to keep the breathing rate at 0.5-1 Hz), and placed in a stereotaxic frame on a heating blanket kept at 37 °C. A craniotomy was made over barrel cortex (1 to 1.5 mm caudal of bregma and 2.5 to 3.1 mm lateral of the midline); through this, a single-shank silicon probe electrode with 16 sites vertically aligned at 50 µm intervals (Neuronexus Technologies Inc., MI, USA) was inserted until the top electrode site was at the surface of the cortex. This allowed us to estimate the distance from the surface of each recording site along the electrode shaft. To allow the electrode track to be reconstructed later, the electrode was dipped in a saturated solution of indocarbocyanine (DiI) in ethanol three times and allowed to dry before use. The tips of between 1 and 4 whiskers were inserted into a fine tube attached to a piezoelectric wafer that bent in response to a 10 ms cosine wave voltage pulse applied across it. Voltage recordings were band-pass filtered (500 Hz-5 kHz), amplified and digitized at 25 kHz. Data acquisition and stimulus production were performed using Brainware (Tucker Davis Technologies, Alachua, USA). This method was adapted for pups from our protocol for adult mice used in Li et al., 2011.

Identification of electrode sites within cortical layer 4. At the end of the recording session, the animal was overdosed with sodium pentobarbitone and perfused with 4% paraformaldehyde. The brain was removed, cryoprotected and sectioned at 40 µm. The depth of each recording site was calculated from the angle of penetration of the electrode (revealed by DiI label) relative to the pial surface (Table S1) and the distance along the electrode shaft (Figure 1B). The location of cortical layer 4 was based on identification of the characteristic stellate cell distribution into “barrels”.

Spike sorting. Action potentials (AP) recorded in each channel were assigned to individual single units on the basis of three criteria: visual inspection of the similarity of the superimposed AP shapes; K-means clustering based on amplitude, width and area of the AP record (Brainware; Tucker Davis Technologies, Alachua, USA); and an auto-correlation histogram with a refractory period of at least 1 ms.

Acute slice preparation

Slices containing the barrel subfield of somatosensory cortex were prepared from C57BL/6 mice (supplied by Harlan, Bicester, UK), ranging from postnatal day (P) 10 to P21 (P30 in Figure S1), according to previously described methods (Agmon and Connors, 1991; Mierau et al., 2004; Rodríguez-Moreno and Paulsen, 2008). Slices were maintained at room temperature (22-27 ºC) until used (1-8 hours) in artificial cerebrospinal fluid containing (in mM): NaCl 126; KCl 3; NaH_2PO_4 1.25; $MgSO_4$ 2, $CaCl_2$ 2, $NaHCO_3$ 26; glucose 10, pH 7.2-7.4; bubbled with carbogen gas (95% O_2/5% CO_2). All recordings were made at room temperature.

Ex vivo electrophysiology

Recording conditions. Slices containing barrels were identified and selected under a stereomicroscope. Two monopolar stimulation electrodes were placed at the base of a barrel (L4). Whole-cell patch-clamp recordings were obtained from L2/3 pyramidal cells in the same barrel column with 5-7 MΩ borosilicate pipettes. The pipette solution contained (in mM): potassium gluconate 110; HEPES 40; NaCl 4; ATP-Mg 4; GTP 0.3, adjusted to pH 7.2 with KOH. In a few experiments CsF replaced potassium gluconate. Cells with a pyramidal-shaped soma were selected for recording using infrared, differential interference contrast optics. All cells were tested for regular spiking responses to positive current injection, characteristic of pyramidal cells. Whole-cell recordings were made using an Axon Multiclamp 700B amplifier (Molecular Devices, Union City, CA). Recordings were low-pass filtered at 2 kHz and acquired at 5 kHz using an ITC-16 acquisition board (Instrutech, Port Washington, NY) and custom-made software procedures programmed in Igor Pro (Wavemetrics, Lake Oswego, OR). All experiments were performed in current clamp ('bridge') mode. Cells were rejected if series resistance changed by more than 15%.

Plasticity experiments. Excitatory postsynaptic potentials (EPSPs) were evoked alternately in two input pathways, test and control, each at 0.2 Hz. After a stable EPSP baseline period of at least 10 minutes, a plasticity protocol was applied to the test pathway. The control pathway was not stimulated during the plasticity protocol. The EPSPs were monitored for 30 minutes after the end of the plasticity protocol. Plasticity was assessed from the slope of the EPSP, measured on the rising phase of the EPSP as a

linear fit between time points corresponding to 25-30% and 70-75% of the peak amplitude during control conditions. For statistical comparisons, the mean EPSP slope was calculated from 60 consecutive sweeps immediately before the start of the plasticity protocol (baseline) and compared with 60 sweeps corresponding to 25-30 minutes after the protocol. Plasticity (potentiation or depression) was expressed as the EPSP slope after the protocol relative to the EPSP slope during baseline.

Replay protocol. Spike times recorded in vivo from layer 4 barrel cortex in response to whisker movement were taken, and only action potentials occurring between 5 and 200 ms after the stimulus were deemed to be event-related. Using software written in-house in Igor Pro, the spike timings from 100 consecutive trials were replayed via electrical stimulation of layer 4 in vitro during conditions of only stimulus-evoked action potentials, spontaneous post-synaptic action potentials (depolarizing current), or no spikes (hyperpolarizing current of 50-120 pA; yielding a hyperpolarization of 5-10 mV without afferent input).

p-LTD protocol. To induce p-LTD, a burst of 3 presynaptic stimuli at 100 Hz was followed 50 ms later by a single stimulus, repeated 100 times. The rest of the procedure and analysis was as described above.

t-LTD protocol. As described in Rodriguez-Moreno and Paulsen (2008).

Protocols in Figure 2. 3S + S: 3 pulses at 100 Hz followed by a single pulse 50 ms later. 2S + S: 2 pulses separated by 10 ms followed by a single pulse 50 ms later. 3S: 3 pulses at 100 Hz not followed by a single stimulus.

Paired pulse protocol. Two stimulation pulses separated by 40 ms were delivered below postsynaptic spike threshold. The slope ratio was calculated as the average of the ratios between the second and the first EPSP slope of 60 responses and plotted before and after the plasticity protocol.

Paired recordings. One pipette was positioned in the barrel (layer 4) and the other in layer L2/3 of the same barrel column. Once double whole-cell patch-clamp configuration was achieved, a brief current pulse was repeatedly applied to the presynaptic cell (layer 4) to check whether presynaptic action potentials could evoke an EPSP in the postsynaptic cell. Only pairs in which spikes in the layer 4 cell evoked monosynaptic EPSPs in the postsynaptic layer 2/3 cell were used. The protocol used to induce LTD was otherwise similar to that used with extracellular stimulation.

Glutamate uncaging. Photolysis of the caged MNI-glutamate was achieved with flashes of focused ultra-violet (UV) light (30 ms, ~120 μm diameter, <460 nm), produced by a 100 W mercury arc lamp (HBO 100, Zeiss in combination with a Uniblitz T123 Driver and LS5 shutter system) connected to the epifluorescence port of the microscope (Axioskop, Zeiss) via a fiberoptic light-guide. MNI-caged glutamate was superfused at a concentration of 100 μM. With an uncaging efficiency of 35% (Canepari et al., 2001), the estimated maximal glutamate concentration during pairing would be 35 μM. Glutamate uncaging produced a transient increase of spontaneous synaptic events in the postsynaptic neuron. Individual uncaging events were therefore separated by 20 s, and the total number of pairings in these experiments was limited to 30.

Drugs. MNI-glutamate, D-AP5, LY341495, FK506 and AM251 were purchased from Tocris Bioscience, Bristol, UK. BAPTA and sodium fluoroacetate were purchased from Sigma, Dorset, UK. All drugs were applied at the start of the recording, except fluoroacetate, which was applied from 20-30 minutes before the start of recording.

Data analysis. Data were analyzed using Igor Pro. Statistical comparisons were made using one-sample, two-sample or paired Student´s *t*-test as appropriate. P-values less than 0.05 were considered significant. Data are presented as mean ± SEM unless otherwise indicated. CV analysis was done on EPSP slopes as described (Rodríguez-Moreno and Paulsen, 2008).

Supplemental References

Agmon, A., and Connors, B.W. (1991). Thalamocortical responses of mouse somatosensory (barrel) cortex in vitro. Neuroscience *41*, 365-379.

Canepari, M., Nelson, L., Papageorgiou, G., Corrie, J.E.T., and Ogden, D. (2001). Photochemical and pharmacological evaluation of 7-nitroindolinyl- and 4-methoxy-7-nitroindolinyl-amino acids as novel, fast caged neurotransmitters. J. Neurosci. Meth. *112*, 29-42.

Li, J., Bravo, D.S., Upton, A.L., Gilmour, G., Tricklebank, M.D., Fillenz, M., Martin, C., Lowry, J.P., Bannerman, D.M., and McHugh, S.B. (2011). Close temporal coupling of neuronal activity and tissue oxygen responses in rodent whisker barrel cortex. Eur. J. Neurosci. *34*, 1983-1996.

Mierau, S.B., Meredith, R.M., Upton, A.L., and Paulsen, O. (2004). Dissociation of experience-dependent and-independent changes in excitatoty synaptic transmission during development of barrel cortex. Proc. Natl. Acad. Sci. USA *101*, 15518-15523.

Rodríguez-Moreno, A, and Paulsen, O. (2008). Spike timing-dependent long-term depression requires presynaptic NMDA receptors. Nat. Neurosci. *11*, 744-745.
